# A Novel Cre Recombinase Mouse Strain for Cell-Specific Deletion of Floxed Genes in Ribbon Synapse-Forming Retinal Neurons

**DOI:** 10.3390/ijms25031916

**Published:** 2024-02-05

**Authors:** Shweta Suiwal, Philipp Wartenberg, Ulrich Boehm, Frank Schmitz, Karin Schwarz

**Affiliations:** 1 Institute of Anatomy and Cell Biology, Department of Neuroanatomy, Medical School, Saarland University, 66421 Homburg, Germany; shwetasuiwal@gmail.com; 2 Institute of Clinical and Experimental Pharmacology, Center for Molecular Signaling (PZMS) and Center for Gender-Specific Biology and Medicine (CGBM), Medical School, Saarland University, 66421 Homburg, Germany; philipp.wartenberg@uks.eu (P.W.); ulrich.boehm@uks.eu (U.B.)

**Keywords:** RIBEYE, retina, photoreceptor, bipolar cells, Cre recombinase, RIBEYE-Cre, ribbon synapse, tau-GFP

## Abstract

We generated a novel Cre mouse strain for cell-specific deletion of floxed genes in ribbon synapse-forming retinal neurons. Previous studies have shown that the RIBEYE promotor targets the expression of recombinant proteins such as fluorescently tagged RIBEYE to photoreceptors and retinal bipolar cells and generates fluorescent synaptic ribbons in situ in these neurons. Here, we used the same promotor to generate a novel transgenic mouse strain in which the RIBEYE promotor controls the expression of a Cre-ER(T2) recombinase (RIBEYE-Cre). To visualize Cre expression, the RIBEYE-Cre animals were crossed with ROSA26 tau-GFP (R26-τGFP) reporter mice. In the resulting RIBEYE-Cre/R26 τGFP animals, Cre-mediated removal of a transcriptional STOP cassette results in the expression of green fluorescent tau protein (tau-GFP) that binds to cellular microtubules. We detected robust tau-GFP expression in retinal bipolar cells. Surprisingly, we did not find fluorescent tau-GFP expression in mouse photoreceptors. The lack of tau-GFP reporter protein in these cells could be based on the previously reported absence of tau protein in mouse photoreceptors which could lead to the degradation of the recombinant tau protein. Consistent with this, we detected Cre and tau-GFP mRNA in mouse photoreceptor slices by RT-PCR. The transgenic RIBEYE-Cre mouse strain provides a new tool to study the deletion of floxed genes in ribbon synapse-forming neurons of the retina and will also allow for analyzing gene deletions that are lethal if globally deleted in neurons.

## 1. Introduction

The vertebrate retina represents a highly organized sensory tissue with a characteristic morphology and clear segregation of retinal neurons (for review, see [1,2,3]). It is built by five principal classes of neurons, i.e., photoreceptors and horizontal, bipolar, amacrine, and retinal ganglion cells. These neurons possess a typical morphology and are connected in a well-characterized manner. Photoreceptors, bipolar and ganglion cells, relay visual information in a centripetal manner from the light-sensitive photoreceptors towards the inner retina and visual brain centers and align their dendritic and axonal processes also along this longitudinal axis. Retinal interneurons, including horizontal cells and amacrine cells, are connected in a lateral direction with their cellular processes.

Synaptic communication between retinal neurons occurs at two distinct, clearly distinguishable synaptic layers, the outer and inner plexiform layers (OPL and IPL, resp.). In the OPL, photoreceptors communicate with bipolar and horizontal cells via specialized glutamatergic ribbon synapses. Ribbon synapses are also used in the IPL to communicate visual signals from bipolar cells to retinal ganglion cells/amacrine cells.

Ribbon synapses are continuously active synapses and are morphologically characterized by the presence of presynaptic specializations referred to as synaptic ribbons [4,5,6]. The synaptic ribbon is a large, electron-dense structure that binds synaptic vesicles and delivers them to the active zone to promote continuous synaptic transmission [7,8,9,10,11].

The RIBEYE protein is the major component of synaptic ribbons [12,13,14,15,16,17]. RIBEYE consists of two main domains, an amino-terminal A domain that is unique to RIBEYE, and a carboxyterminal B domain that is identical, except for the first 20 amino-terminal amino acids, with the transcriptional co-repressor CtBP2 [12]. The *RIBEYE/CtBP2* gene is a bi-functional gene and generates two gene products [18]. The CtBP2 promotor drives ubiquitous CtBP2 expression in most eukaryotic cells [18,19,20,21], whereas the RIBEYE promotor drives RIBEYE expression only in ribbon synapse-forming cells [12,22,23,24,25]. 

The RIBEYE promotor is well suited to direct expression to ribbon synapse-forming cells in mice [25]. A Cre mouse strain that is controlled by the RIBEYE promotor could thus be very useful to delete protein expression in ribbon synapse-forming neurons in conditional knockout models in which the corresponding alleles are flanked by *loxP* sites. In the present study, we generated such a mouse model and characterized its functionality in the mouse retina.

## 2. Results

**Figure 1:**
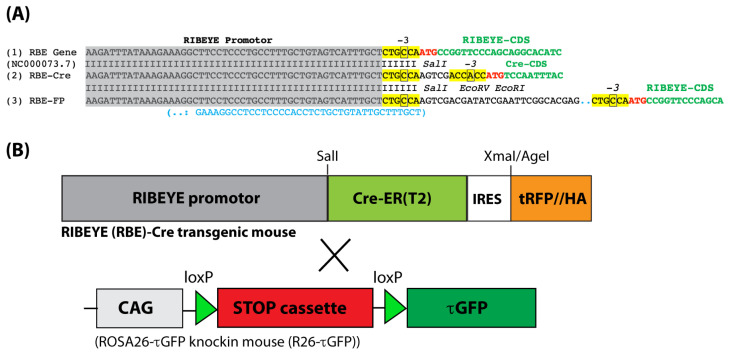
Genomic maps. (**A1**) Schematic depiction of part of the mouse RIBEYE gene. The grey box shows the RIBEYE promotor. The highlighted, yellow boxed nucleotide sequence shows the Kozak sequence. ATG printed in red indicates the start ATG. Nucleotides depicted in green show the RIBEYE protein coding sequence (CDS). (**A2**) Schematic map of the mouse RIBEYE-Cre transgenic construct aligned to the mouse RIBEYE gene from A1. Grey box, RIBEYE promotor; yellow boxed nucleotide sequence, Kozak sequence. The indicated SalI restriction site was used for cloning the Cre transgene (see also (**B**)). The ATG printed in red represents the start ATG codon; nucleotides in green indicate the CDS of the Cre recombinase. (**A3**) For comparison, the genomic map of the previously published RIBEYE-FP (RBE-FP) [25] mouse is also shown. (**B**) Schematically depicts the applied mouse breeding schedule. RIBEYE-Cre mice were crossed with R26-τGFP mice [27,28]. Cre expression from the RIBEYE-Cre/R26-τGFP mouse was visualized by tau-GFP fluorescence signals after excision of the STOP cassette from the ROSA26 τGFP allele. Abbreviation: CDS, coding sequence.

In a previous study [25], we showed that the mouse RIBEYE promotor specifically drives the expression of fluorescently tagged RIBEYE protein in photoreceptors and bipolar cells to generate fluorescent synaptic ribbons in the living retina. Photoreceptors and bipolar cells are the principal neurons that form ribbon synapses in the retina. To establish a tool that can be used to selectively delete floxed genes only in ribbon synapse-forming neurons, we used the same promotor to drive the expression of Cre-ER(T2), a tamoxifen-inducible Cre recombinase [26]. A consensus Kozak sequence was placed upstream of the Cre-ER(T2) coding sequence (CDS) in the transgenic construct (Figure 1A). 

The RIBEYE-Cre mice were then crossed with R26-τGFP reporter mice to visualize Cre activity (Figure 1B). In the R26-τGFP mouse, fluorescent tau-GFP is expressed only after removal of a floxed, transcriptional genomic STOP cassette by a Cre recombinase [26,27,28,29]. Upon expression, tau-GFP binds to microtubules to produce fluorescently tagged microtubules [27,28]. The microtubule system in the retina is well known [30,31,32,33]. For transgene expression, we analyzed the resulting tau-GFP-positive, Cre-positive mice (RIBEYE-Cre/R26-τGFP mice) for immunosignals by using cryosections from the respective retinas. Cryosections from Cre-negative, tau-GFP-positive littermate mice (R26-τGFP mice) served as negative controls. 

**Figure 2:**
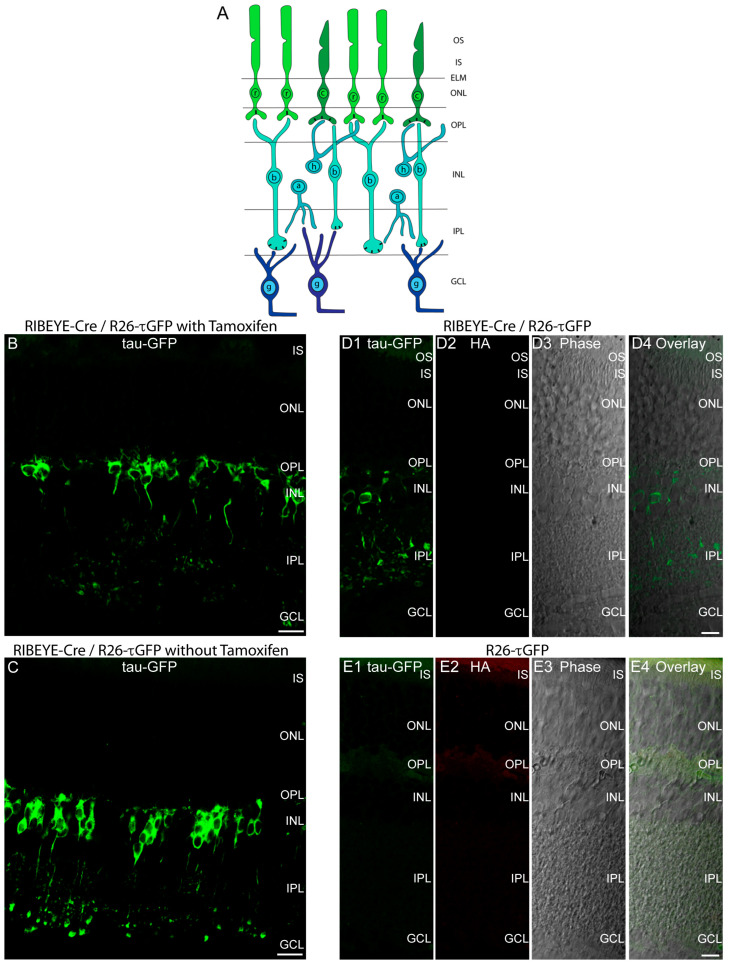
(**A**) Schematic drawing of retinal layers. (**B**,**C**) A 10 µm thick cryostat section from Cre-positive, τGFP-positive heterozygous RIBEYE-Cre/R26-τGFP mice that were injected with tamoxifen (**B**) or were control-injected ((**C**), no tamoxifen). Strong tau-GFP fluorescence was detectable in retinal bipolar cells that have their cell bodies in the INL. (**D1**–**D4**,**E1**–**E4**) A 10 µm thick cryostat section from Cre-positive, τGFP-positive heterozygous RIBEYE-Cre/R26-τGFP mice ((**D1**), non-injected) or from Cre-negative, τGFP-positive heterozygous R26-τGFP mice ((**E1**), non-injected) immunolabeled with antibodies against HA. No HA signal or RFP signal is visible in the red channel (**D2**,**E2**) indicating that the IRES sequence is not working in the transgenic mouse. (**D3**,**E3**) The phase contrast micrographs. In (**D4**,**E4**) all corresponding channels were overlaid. (**B**,**C**) Maximum projections; (**D1**–**D4**,**E1**–**E4**) show single confocal planes. Abbreviations: OS, outer segment; IS, inner segments; ELM, external limiting membrane; ONL, outer nuclear layer; OPL, outer plexiform layer; INL, inner nuclear layer; IPL, inner plexiform layer; GCL, ganglion cell layer; r, rod; c, cone; b, bipolar cell; h, horizontal cell; a, amacrine cell; g, ganglion cell. Rectangular black bars in the presynaptic terminals represent synaptic ribbons. Scale bars: 5 µm.

First, we checked for the tamoxifen-inducibility of the Cre-ER(T2) by comparing cryosections from the retinas of tamoxifen-injected and non-tamoxifen-injected mice. Retinal layers are schematically depicted in Figure 2A. Retinas from both tamoxifen- and non-tamoxifen-injected mice showed a similar fluorescence pattern with a strong labeling of retinal bipolar cells (Figure 2B,C). These data indicate that the expression of Cre-ER(T2) is “leaky” because Cre recombinase is already active in the non-injected RIBEYE-Cre/R26 τGFP mice as judged by tau-GFP protein expression (Figure 2C). A leaky activity of Cre-ER(T2) has already previously been observed, i.e., that Cre-ER(T2) is already active without tamoxifen injection [34,35,36,37]. The fluorescent tau-GFP labeling of retinal bipolar cells as shown in Figure 2(B–D1) is specific because it was absent in Cre-negative mice (Figure 2(E1)). Photoreceptors in the outer nuclear layer (ONL) of the outer retina (Figure 2A) did not show a tau-GFP signal (Figure 2B–E; see also below). In the transgenic vector, an IRES site followed by an HA-tag and a tag-RFP (tRFP) was placed downstream (3′) of the Cre-ER(T2) recombinase. This IRES site turned out to be non-functional because the tags driven by the IRES site did not produce any fluorescence signal (Figure 2(D2,D4,E2,E4)). The red channel did not show a signal after immunolabeling with anti-HA, nor a direct endogenous red immunofluorescence signal as it would be provided by the tRFP tag (Figure 2(D2,E2)). 

**Figure 3:**
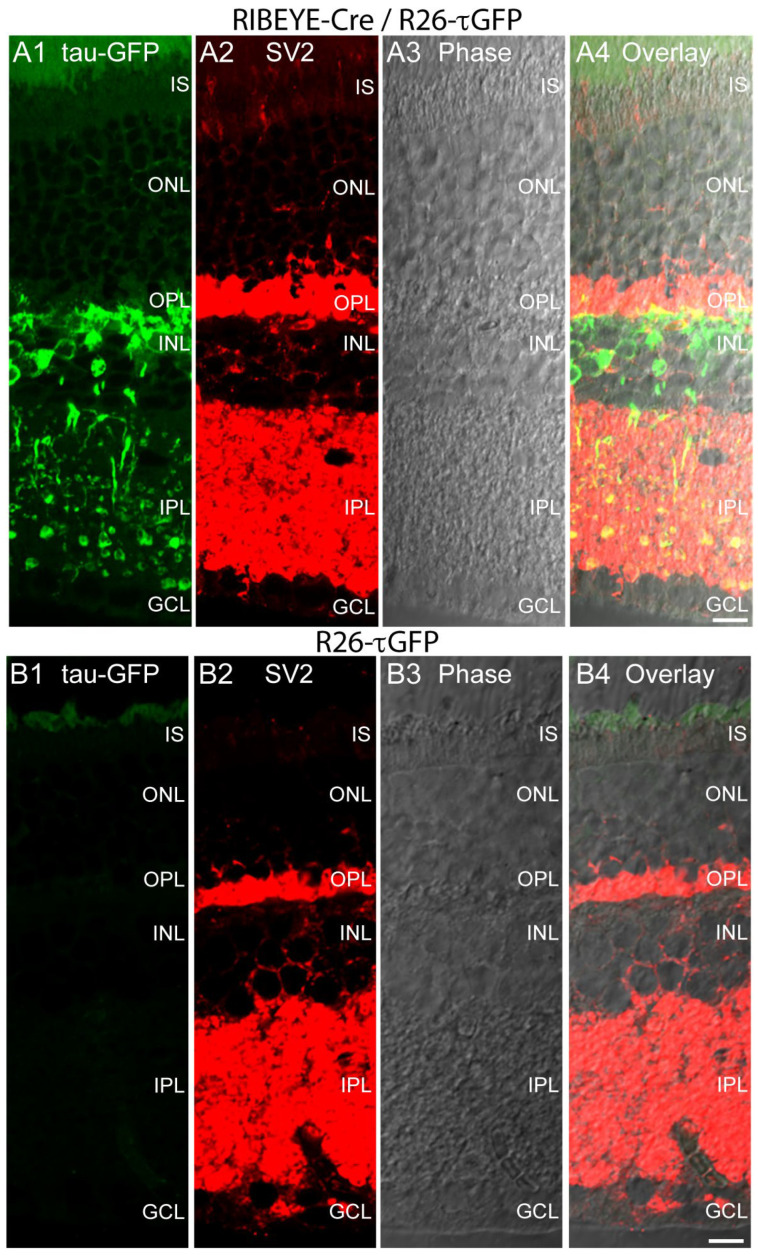
(**A1**–**A4**,**B1**–**B4**) A 10 µm thick cryostat section from Cre-positive, τGFP-positive heterozygous RIBEYE-Cre/R26-τGFP mice (**A1**–**A4**) or from Cre-negative, τGFP-positive heterozygous R26-τGFP mice (**B1**–**B4**) immunolabeled with mouse monoclonal antibodies against SV2. In the green channel, the tau-GFP fluorescence is shown. (**A3**,**B3**) The phase contrast micrographs. In (**A4**,**B4**) all channels were overlaid. Images are maximum projections from z-stacks. Samples were from mice that were not injected with tamoxifen because Cre-ER(T2) activity in the RIBEYE-Cre mice was found to be “leaky” (tamoxifen-independent) (see Figure 2). Abbreviations: IS, inner segments; ONL, outer nuclear layer; OPL, outer plexiform layer; INL, inner nuclear layer; IPL, inner plexiform layer; GCL, ganglion cell layer. Scale bars: 5 µm.

We further characterized the expression pattern of tau-GFP-positive cells in the RIBEYE-Cre/R26-τGFP animals by immunolabeling with an antibody against the synaptic vesicle protein SV2 (anti-SV2) (Figure 3). Anti-SV2 immunolabeling provided strong immunolabeling of the two synaptic layers of the retina, the OPL, and IPL (Figure 3(A2,B2)). The tau-GFP-positive retinal cells in the inner retina displayed a bipolar cell-typical distribution (Figure 3(A1)). They extended between the outer plexiform layer (OPL) and inner plexiform layer (IPL) that were immunolabeled with SV2 (Figure 3(A2,B2)). The tau-GFP-labeled cells extended from the OPL to the IPL (Figure 3(A1)) as expected by the morphology of the retinal bipolar cells. The dendrites of the retinal bipolar cells receive their input at the photoreceptor synapses in the OPL and send their axons into the IPL in which they synaptically connect to retinal ganglion cells (RGCs) and amacrine cells (ACs). Again, tau-GFP was present only in Cre-positive retinas (Figure 3(A1)) but not in Cre-negative retinas (Figure 3(B1)) whereas the SV2 immunolabel was present in both genotypes in an identical, undistinguishable manner (Figure 3(A2,B2)).

**Figure 4:**
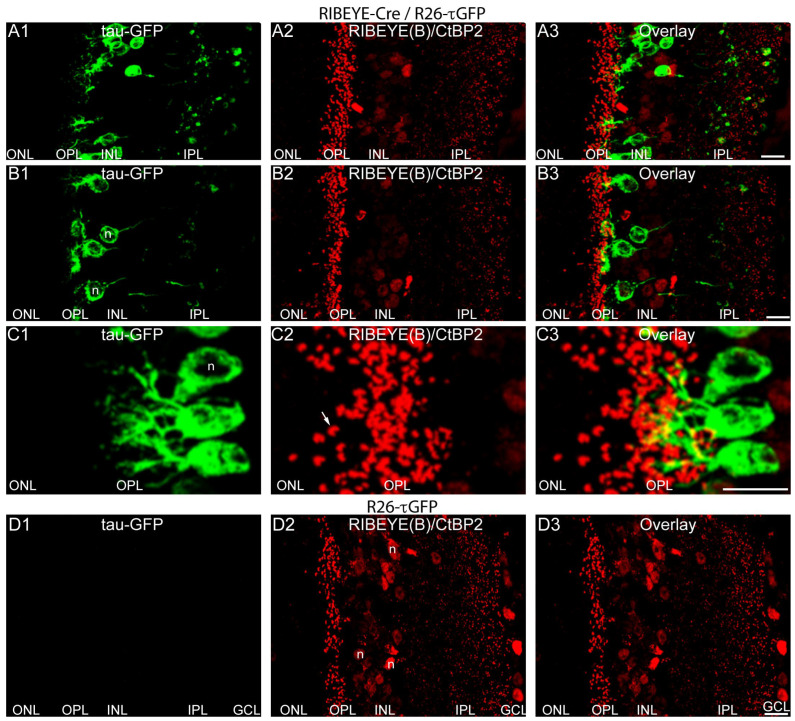
(**A1**–**D3**) A 10 µm thick cryostat section from Cre-positive, τGFP-positive heterozygous RIBEYE-Cre/R26-τGFP mice (**A1**–**A3**,**B1**–**B3**,**C1**–**C3**) or from Cre-negative, τGFP-positive heterozygous R26-τGFP mice (**D1**–**D3**) immunolabeled with mouse monoclonal antibodies against RIBEYE B domain/CtBP2 (clone 2D9). In the green channel (**A1**,**B1**,**C1**,**D1**), the tau-GFP fluorescence is shown. (**A2**,**B2**,**C2**,**D2**) The RIBEYE immunosignals. In (**A3**,**B3**,**C3**,**D3**) green and red channels are overlaid. Images are maximum projections from z-stacks. The white arrow in (**C2**) indicates a single immunolabeled synaptic ribbon. Samples were from mice that were not injected with tamoxifen because Cre-ER(T2) activity in the RIBEYE-Cre mice was found to be “leaky” (tamoxifen-independent) (see Figure 2). Abbreviations: IS, inner segments; ONL, outer nuclear layer; OPL, outer plexiform layer; INL, inner nuclear layer; IPL, inner plexiform layer; GCL, ganglion cell layer; n, nucleus. Scale bars: 5 µm.

Also, double immunolabeling with antibodies against RIBEYE (Figure 4(A2,B2,C2,D2)), the main component of synaptic ribbons [12,13], showed that the tau-GFP-positive retinal cells reached their dendrites towards synaptic ribbons in the OPL and extended their axons into the inner plexiform layer as it is typical for retinal bipolar cells (Figure 4(A1,A3,B1,B3,C1,C3)). Figure 4(D1–D3) show cryosections from Cre-negative, τGFP-positive R26-τGFP mice (littermate negative control) immunolabeled with anti-RIBEYE antibodies. The RIBEYE immunosignal in the R26-τGFP-negative control mice (Figure 4(D2)) is undistinguishable from the RIBEYE immunosignals in Cre-positive, τGFP-positive RIBEYE-Cre/R26-τGFP mice (Figure 4(A2,B2,C2)). 

In contrast, tau-GFP fluorescence signals are completely absent from Cre-negative control retinas (Figure 4(D1)). Please note that the antibody against the RIBEYE B domain used for immunolabeling (clone 2D9, [38]) not only detects RIBEYE but also the nuclear co-repressor CtBP2. As mentioned in the introduction, the RIBEYE B domain is virtually identical to CtBP2 except for the first 20 amino acids [12]. Therefore, the cryo-sections immunolabeled with antibodies against the RIBEYE B domain not only show the immunolabeled synaptic ribbons but also a weak nuclear staining caused by the presence of CtBP2 (Figure 4(A2,B2,D2)); exemplary immunolabeled nuclei are annotated in Figure 4(D2).

**Figure 5:**
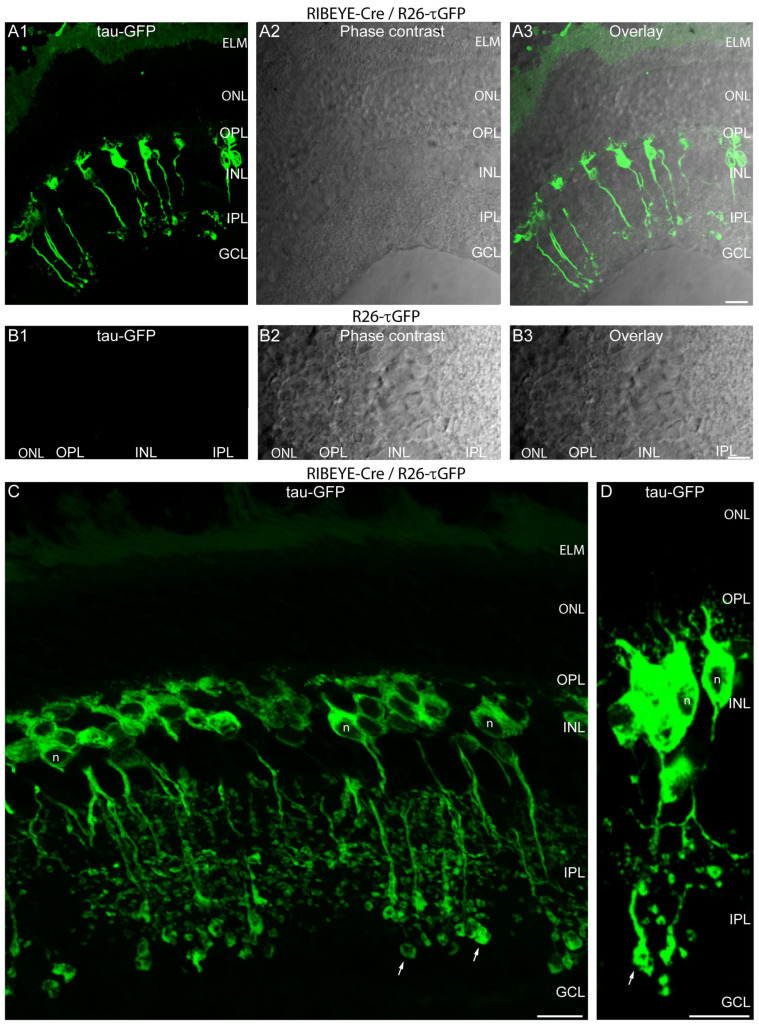
(**A1**–**B3**) A 10 µm thick cryostat section from Cre-positive, τGFP-positive heterozygous RIBEYE-Cre/R26-τGFP mice (**A1**–**A3**) or from Cre-negative, τGFP-positive heterozygous R26-τGFP mice (**B1**–**B3**). In the green channel (**A1**,**B1**), the tau-GFP fluorescence is shown. (**A3**,**B3**) The phase contrast micrographs. In (**A3**,**B3**), both channels are overlaid. (**C**,**D**) High magnification images of retinas and individual fluorescent retinal bipolar cells (maximum projections of z-stacks) from Cre-positive, R26-τGFP-positive (RIBEYE-Cre/R26-τGFP) mice. The white arrows in (**C**,**D**) indicate presynaptic terminals of retinal bipolar cells in the IPL. Samples were from mice that were not injected with tamoxifen because Cre-ER(T2) activity in the RIBEYE-Cre mice was found to be “leaky” (tamoxifen-independent) (see Figure 2). Abbreviations: IS, inner segments; ONL, outer nuclear layer; OPL, outer plexiform layer; INL, inner nuclear layer; IPL, inner plexiform layer; GCL, ganglion cell layer; n, nucleus. Scale bars: 5 µm.

Higher magnification images of cryosections from Cre-positive, τGFP-positive RIBEYE-Cre/R26-τGFP mice confirmed the typical morphology of retinal bipolar cells (Figure 5(A1,A3,C,D)). The dendrites of the retinal bipolar cells extended into the outer plexiform layer (OPL) and the axonal processes ended in the inner plexiform layer (IPL) (Figure 5(A1,A3,C,D)). Many of the axons terminated in the ON sub-layer of the IPL close to the retinal ganglion cells (RGCs). Figure 5(B1–B3) demonstrate the absence of tau-GFP fluorescence in Cre-negative, τGFP-positive littermate control mice. 

In conclusion, these data clearly show that Cre recombinase is expressed in retinal bipolar cells as judged by the strong tau-GFP fluorescence in these retinal neurons. Surprisingly, photoreceptors that have their cell body in the ONL did not show tau-GFP fluorescence in the very same sections in which the retinal bipolar cells were strongly tau-GFP-positive. This finding was surprising because we showed in a previous study [25] that the same RIBEYE promotor, as used in the present study, controls expression both in photoreceptors and retinal bipolar cells. 

**Figure 6:**
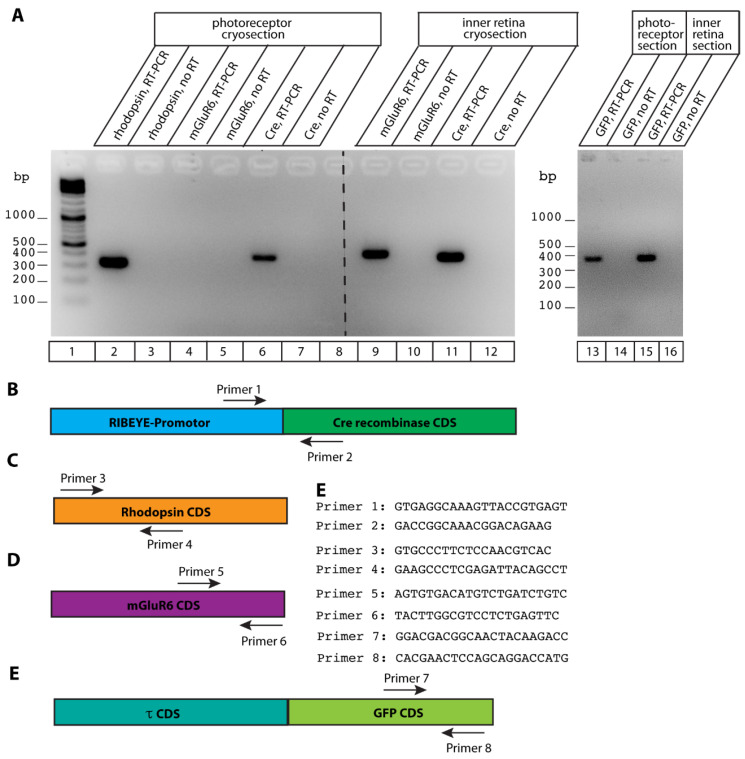
(**A**) Agarose gel showing results of RT-PCR reactions with the indicated primers. PCR reactions from samples without reverse transcription served as controls for genomic DNA contamination. Cryosections from the photoreceptor layer were positive for rhodopsin mRNA (positive control demonstrating presence of rod photoreceptors in the respective section, lane 2), Cre-recombinase (lane 6), and τ-GFP mRNA (lane 13). mGluR6 mRNA, as a marker mRNA for invaginating ON bipolar cells [39,40,41], was absent in the photoreceptor section, demonstrating that the photoreceptor section is not contaminated by inner retina neurons, e.g., retinal bipolar cells. mGluR6 is positive in the inner retina demonstrating that the primer pairs and PCR conditions are principally working (positive control for mGluR6 reaction, lane 9). As expected, Cre mRNA and τ-GFP mRNA are also expressed in the inner retina (lane 11, lane 15), as independently shown with the reporter mice by immunofluorescence microscopy with the ROSA26 τGFP reporter mouse (Figure 2, Figure 3, Figure 4 and Figure 5). All PCR reactions without reverse transcription were negative (lanes 3, 5, 7, 10, 12) demonstrating that the samples were not contaminated by genomic DNA. (**B**–**E**) Schematically depicted location of the primers of the RT-PCR reactions in the RIBEYE-Cre-ER(T2) transcript (**B**), the rhodopsin transcript (**C**), the mGluR6 transcript (**D**), and the τGFP transcript (**E**). Abbreviation: CDS, coding sequence.

Since fluorescent tau protein was not obviously expressed in mouse photoreceptors, we tested whether the Cre-ER(T2) transcript is detectable in mouse photoreceptors by RT-PCR. For this purpose, we performed horizontal, “layer-wise” cryo-sectioning of Cre-positive, τGFP-positive mouse retina. We obtained cryosections from the outer retina that contained rhodopsin mRNA (Figure 6A, lane 2) but not mGluR6 mRNA (Figure 6A, lane 4) indicating that we successfully obtained cryosections from rod photoreceptors that were not contaminated by the inner retina. mGluR6 is expressed in invaginating rod bipolar cells [39,40,41]. Photoreceptor slices contained Cre-ER(T2) mRNA as judged by RT-PCR (Figure 6A, lane 6) indicating that Cre is expressed in photoreceptors of RIBEYE-Cre transgenic mice. Unfortunately, the Cre antibodies available to us were not sensitive enough to detect Cre expression in photoreceptor slices by immunofluorescence microscopy. 

We found tau-GFP mRNA not only in the inner retina that contains the bipolar cells (Figure 6, lane 15) but also in the photoreceptors (Figure 6, lane 13) as judged by RT-PCR. Therefore, the reason for the absence of fluorescent tau-GFP in photoreceptors could be that tau protein is not stable in photoreceptors and becomes degraded in photoreceptors (see discussion, Section 3). 

## 3. Discussion

In a previous study, we showed that the RIBEYE promotor specifically drives the expression of recombinant, fluorescently tagged RIBEYE protein to produce fluorescent synaptic ribbons in retinal photoreceptors and bipolar cells [25]. These retinal neurons are the neurons that generate ribbon synapses in the retina [25]. In the present study, we used the same promotor to generate a novel Cre mouse line under the control of the RIBEYE promotor. The purpose of this attempt was to generate a novel transgenic mouse to drive the expression of tamoxifen-inducible Cre-ER(T2) recombinase in ribbon synapse-forming neurons. Such a mouse model would be relevant for the ribbon synapse community, e.g., to study the effects of the deletion of floxed synapse-relevant genes in ribbon synapses. It would also allow for analyzing the deletion of genes that are lethal if globally deleted in all neurons. The retina is a well-established system to analyze synaptic processes, e.g., at the rod bipolar to amacrine AII synapse [13,42] in the inner plexiform layer or at photoreceptor synapses in the outer plexiform layer [43,44,45,46]. 

To monitor Cre expression, we crossed the RIBEYE-Cre mice with R26-τGFP knockin mice in which tau-GFP protein is expressed only after the Cre-mediated excision of a transcriptional STOP cassette. These mice showed a strong expression of tau-GFP in retinal bipolar cells. Retinal bipolar cells are one major type of retinal neurons that form ribbon synapses [6]. Most of the fluorescent bipolar cells terminated in the ON sublayer of the inner plexiform layer. This is expected because the mouse retina is a rod-dominated retina and rod photoreceptors predominantly feed their signals to invaginating rod ON bipolar cells that terminate into the ON sub-lamina of the IPL [47,48,49,50,51,52,53,54,55]. Tau-GFP expression occurred without tamoxifen injection indicating that the activity of the Cre recombinase is “leaky” and active already without tamoxifen. The tamoxifen-independent activity of Cre-ER(T2) has already been previously described [34,35,36,37].

Surprisingly, we did not observe tau-GFP fluorescence in photoreceptors of Cre-positive, τGFP-positive mice although photoreceptors from these mice clearly do form ribbon synapses as shown by immunolabeling with antibodies against RIBEYE (Figure 4). The RIBEYE immunolabeling of the transgenic mice is very similar to previously published immunolabeling data, clearly demonstrating that ribbon synapses are also made by photoreceptor synapses in the transgenic mice. Several reasons could apply to this phenomenon. For us, the most likely reason for the absence of tau-GFP protein in mouse photoreceptors is the previously reported absence of tau protein from mouse photoreceptors [56,57,58,59]. Therefore, heterologous expressed recombinant tau protein might also be unstable in mouse photoreceptors and subsequently degraded. In favor of such a possibility, we found the presence of Cre-mRNA in cryosections containing photoreceptors that were devoid of mRNA signals from the inner retina, e.g., mGluR6 mRNA from ON bipolar cells. Furthermore, we were able to detect τ-GFP mRNA in these photoreceptor-containing slices, indicating successful excision of the transcriptional STOP cassette in the mouse photoreceptors. Future investigations in which the RIBEYE-Cre mice will be bred, e.g., with ROSA26 reporter mice other than R26-τGFP could address this possibility of protein instability. In species other than mice (e.g., humans, cows, drosophila), species-dependent expression patterns for tau protein were reported [33,57,60,61,62]. 

We cannot completely exclude that additional factors could also play a role. As mentioned in the methods part, we placed a consensus Kozak sequence upstream of the start ATG of the Cre coding sequence. We expected that this would help to improve the efficiency of correct translational initiation of the corresponding mRNA for which the Kozak sequence is important ([63]; for review, [64]). The RIBEYE gene has a non-standard Kozak sequence at the -3 nucleotide position of the Kozak sequence (Figure 1A). The -3 nucleotide position is considered to be crucial for the function of the Kozak sequence in translational initiation [63,65,66,67,68]. The -3 position in the RIBEYE gene of the mouse is occupied by a pyrimidine (Figure 1A) instead of a purine (A/G) in the optimal Kozak consensus sequence [63,65,66,67,68]. In principle, we cannot exclude that this consensus Kozak sequence is less efficiently recognized by the ribosomal machinery of photoreceptors than by the translational machinery of retinal bipolar cells. But we believe that this possibility is not very likely. Cell-specific, Kozak sequence-dependent differential efficiency of translational initiation has not been previously reported so far to our knowledge. Therefore, the absence of tau-GFP in Cre-positive mouse photoreceptors is more likely based on the instability of tau-GFP in mouse photoreceptors that normally do not express tau protein. Since Cre-mRNA and τ-GFP mRNA are clearly present in photoreceptors of transgenic RIBEYE-Cre mice (Figure 6), we would predict that the RIBEYE-Cre mouse will be also useful for gene deletion in photoreceptors. But this remains to be shown by future investigations. Ribbon synapses are also made in the inner ear and the pineal gland [5,6]. Therefore, this novel Cre mouse could be also interesting for researchers in this field.

## 4. Materials and Methods

### 4.1. Mice/Ethical Considerations

All animal procedures, including general animal care procedures, anesthesia, and sacrificing mice for organ collection were reviewed and approved by the local animal authorities (Tierschutzbeauftragte der Universität des Saarlandes and Landesamt für Verbraucherschutz; Geschäftsbereich 3; 66115 Saarbrücken, Germany; GB 3-2.4.2.2-25-2020). Only adult, mature mice (age range between 20 and 50 weeks) of both sexes were used for the experiments. Cre-positive, τGFP-positive heterozygous mice (denoted as RIBEYE-Cre/R26-τGFP mice) were used as experimental animals; Cre-negative, τGFP-positive heterozygous littermate mice (denoted as R26-τGFP mice) served as controls. The results of the morphological experiments were assembled from 4 pairs of RIBEYE-Cre/R26-τGFP mice and the respective R26-τGFP littermate control mice. 

#### ROSA26-CAGS-τGFP Mice

Tau (τ) is a microtubule-associated, microtubule-binding protein [69,70,71,72]. The ROSA26-τGFP knockin (KI) mice contain a tau-GFP expression cassette [27] in the ROSA26 genomic locus that controls expression of green fluorescent protein-tagged tau protein (tau-GFP) after Cre-mediated excision of a transcriptional STOP cassette [28]. tau-GFP fluorescently labels microtubules and thus allows identification of cells in which Cre has been expressed [27,28]. Genotyping of the recombinant τGFP allele in the ROSA26 locus was performed using a WT/KI ROSA26 forward primer GGAAGCACTTGCTCTCCCAAAG common for the wild-type and knock-in PCR reaction. The reverse primer for the detection of the τGFP KI allele was GGGCGTACTTGGCATATGATACAC; the reverse primer for the wild-type allele was CTTTAAGCCTGCCCAGAAGACTC. The resulting PCR reactions lead to product lengths of 256 bp for the wild-type allele and 495 bp for the R26-τGFP KI allele. 

### 4.2. Cloning of the Transgenic Vector and Generation of Transgenic RIBEYE-Cre Mice

The RIBEYE promotor controls expression of RIBEYE protein specifically in retinal neurons that form ribbon synapses, i.e., photoreceptors and bipolar cells, as previously shown [25]. In the present study, we used the same RIBEYE promotor to generate a transgenic tamoxifen-inducible Cre-ER(T2) mouse line that drives Cre expression under the control of the RIBEYE promotor. For the cloning of the transgenic vector, the RIBEYE-tRFP transgenic vector [25] served as backbone. From this vector, RIBEYE cDNA was excised with SalI and AgeI and replaced by a Cre-ER(T2)-IRES2 cassette. In short, IRES2 site was PCR-amplified from pIRES2-DsRed (Takara) using the 5′ forward primer TTTGAATTCACCGGTGCCCCTCTCCCTCCCCCCCCC and the 3′ reverse primer TTTCCCGGGTTGTGGCCATATTATCATCGTGTTTTTC and cloned into EcoRI/XmaI sites of pBluescript KS (pSK-IRES2). The open-reading frame of Cre-ER(T2) [26,73] was PCR-amplified with forward primer (TTTGTCGACCACCATGTCCAATTTACTG) and reverse primer (TTTCCCGGGTCATCAGACTGTGGCAGG) and cloned into SalI/AgeI sites of pSK-IRES2 to generate the vector pSKCreERT2-IRES2. From this vector, the Cre-ER(T2)-IRES2 cassette was excised with SalI/XmaI and cloned into SalI/AgeI digested RIBEYE-tRFP transgenic vector [25]. All cloning steps and the final transgenic vector were verified by sequencing. 

The transgenic construct was excised via the flanking SacII and NotI sites and gel-purified prior to pronucleus injection, which was performed by F. Zimmermann/S. Dlugosz (IBF; University of Heidelberg). Positive founder animals were identified via PCR using the following primer pairs: F1 (AGTTGTTTGTTCCTGCCGTCTC, in promotor), R1 (TGGCCCAAATGTTGCTGGATAG, in Cre); F2 (TCCTCATCCTCTCCCA CATCAG, in ER-(T2)), R2 (TGCTTCCTTCACGACATTCAACAG; in IRES site); F3 (TCCAAGAAA CCCGCTAAGAACC, in tRFP), R3 (GTGAAAGTAGGCGTTGAGCCAG, in SNAP tag). The transgene placed behind the IRES site was not functional and did not lead to any detectable expression of the tags controlled by the IRES site, i.e., tRFP and HA. Transgenic mice were genotyped for Cre recombinase using forward primer GGTTTCCCGCAGAACCTGAA and reverse primer AGCCTGTTTTGCACGTTCACC and genomic DNA was isolated as described [13,25]. A Cre-positive PCR reaction generates a PCR product of 215 bp. 

### 4.3. Methods

#### 4.3.1. Tamoxifen Injection

Tamoxifen injection was performed largely as previously described [74]. Tamoxifen (Thermo Scientific, Dreieich, Germany; #J63509.03) was dissolved in Miglyol 812 (Caesar & Lorentz GmbH, Hilden Germany; #CSLO3274.1000) at a concentration of 10 mg/mL. For induction of Cre-ER(T2), mice were either injected with tamoxifen or solvent alone. Each mouse of the experimental group received four consecutive intraperitoneal tamoxifen injections, one per day, at a dose of 100 mg/kg body weight. Control mice were injected using the same schedule with Miglyol 812. 

#### 4.3.2. Cryo-Sample Preparation and Cryo-Sectioning of Transgenic Mouse Eyes

Mice were anesthetized with isoflurane and killed by cervical dislocation under ambient light. Mouse eyes were enucleated within 5 min post mortem. The anterior eyecup and the lens were removed after a circular cut along the equator of the eye. The posterior eyecup with the attached retina was immersion-fixed with 4% freshly depolymerized paraformaldehyde in PBS (overnight, 4 °C). Next, the posterior eyecups were washed with PBS (5 × 5 min) and infiltrated with 30% sucrose in PBS (overnight, 4 °C) for cryo-protection. The cryo-protected tissues were then flash-frozen in liquid nitrogen-cooled isopentane, as previously described [12,75]. From these samples, 10 μm thick cryostat sections were cut with a Leica cryostat CM950, collected on gelatinized glass cover slides, heat-fixed on a heating pad (60 °C, ~15 min), and stored at −20 °C until use. Gelatinization of glass cover slides was performed by treating the glass cover slides with a solution containing 1% gelatin and 0.1% KCr(SO_4_)_2_ in H_2_O. Gelatin was first dissolved in H_2_O by gentle heating (~40 °C) before the KCr(SO_4_)_2_ was added. Solution was filtered before use. Glass cover slides were dipped twice in this solution and then air-dried.

#### 4.3.3. Immunohistochemistry of 10 μm Thick Cryostat Sections of Mouse Retinas

Cryostat sections obtained as described above were heat-fixed on a heating pad (~60 °C) for ~15 min. After removal from the heating pad and equilibration to room temperature, cryo-sections were incubated with 0.5% BSA in PBS (denoted as blocking buffer in the manuscript) for blocking unspecific protein binding sites on the sections (~30 min, RT). Incubation of the heat-fixed cryostat sections with primary and secondary antibodies, and negative and positive controls was performed as previously described above [12]. Primary antibodies were diluted in blocking buffer. Dilutions and references of the primary antibodies are given in Table 1. Incubation in the primary antibody solutions was performed overnight (at 4 °C). After several washes with PBS, sections were incubated for 1 h with the indicated secondary antibodies. Secondary antibody dilutions and references are given in Table 2. Following several washes with PBS, sections were embedded in NPG antifade, as previously described [12,17,38,75,76]. Immunolabeled sections were analyzed by confocal microscopy with an A1R confocal microscope, see below. 

#### 4.3.4. Confocal Microscopy

Confocal microscopy was performed with an A1R confocal microscope (Nikon, Düsseldorf, Germany) operated as previously described [17,38,75,76]. Images were acquired with a 60x/1.40 N.A. oil objective and he 488 nm and 568 nm laser excitation lines. Image acquisition was performed with the NIS Elements software (NIS Elements AR 3.2, 64 bit; Nikon, Düsseldorf, Germany). Confocal z-stacks were acquired as previously described [17] and presented as maximum projections, as indicated in the respective figures. 

#### 4.3.5. Horizontal Cryo-Sectioning of the Mouse Retina

Horizontal (“retinal layer-wise”) cryo-sectioning of the mouse retina was performed similarly as previously described [81,82]. For sectioning of horizontal cryo-sections that contained photoreceptors free of the inner retinal layers, eyes from Cre-positive, tau-GFP heterozygous mice were enucleated within 5 min post-mortem. The isolated eyes were poked with a 21 G needle at the equatorial plane and the anterior eyecup was removed with a circular cut along the equatorial plane. Next, the retina was gently peeled off from the posterior cup in sterile PBS after dissection of the retina from the lamina cribrosa. Small cuts were made on four opposing sites of the retina so that the retina could be flat-mounted onto black-gridded nitrocellulose filter membranes (Millipore, Darmstadt, Germany; #HABG01300) with the ganglion cell side facing the nitrocellulose membrane. The membranes with the attached retina were cut into a small square size to keep the retina in a flat horizontal position. Gentle suction was applied on membrane filter with the attached retina by putting it in a silica sieve funnel attached to a syringe to further strengthen the attachment of the retina to the filter. The membrane-attached retina was placed onto a plastic coverslip (Thermanox plastic coverslips, NUNC, Dreieich, Germany). The coverslip with attached nitrocellulose membrane and retina was rapidly submerged into melting liquid nitrogen-cooled isopentane for flash-freezing and subsequently directly into liquid nitrogen. Cryosections from the frozen samples were prepared with a Leica cryostat (Leica CM 3050 S). The coverslip with nitrocellulose filter and retina was attached to the cryostat holder using OCT medium (Neg-50, Epredia; Fisher Scientific, Schwerte, Germany). The cutting stage of the sectioning knife was carefully aligned parallel to the coverslip for horizontal sectioning of the retina. Five consecutive sections (10 µm thick) were cut from the outer retina and the remaining inner retina was also collected. Each section of the outer retina and inner retina were immediately placed in 1.5 mL reaction tubes containing 300 µL SKP buffer of the RNA/DNA/Protein Purification Kit (Norgen via BioCat, Heidelberg, Germany, 47700). 

#### 4.3.6. RNA and Protein Extraction from Retinal Cryo-Sections

RNA/protein was extracted with an RNA/DNA/Protein Purification Kit (Norgen Biotek via BioCat, Heidelberg, Germany, 47700) according to the manufacturer’s instruction manual. One single cryosection from outer part of retina and one single cryosection from the inner part of retina were added separately to 300 µL guanidium salt containing SKP-lysis buffer (provided with the kit). An amount of 3 µL β-mercaptoethanol was added to buffer before lysis and left on ice for 30 min. The tissue lysates were loaded onto the gDNA purification column to remove genomic DNA and were centrifuged at 8000 rpm for 2 min. The flow-through from the column was retained for RNA purification as it contains RNAs and proteins. RNA was precipitated by adding 180 µL 96–100% ethanol to 300 µL flow-through, subsequently loaded onto the RNA/protein purification column, and centrifuged at 6000 rpm for 2 min. The RNA column flow-through was retained for protein purification and the column was washed with wash solution A. Thereafter, on-column DNA removal was performed at 30–37 °C by adding 100 μL of the RNase-free DNase I to remove residual genomic DNA. The RNA column was washed with wash solution A and total RNA was eluted in 30 µL RNA elution buffer by centrifugation at 2000 rpm (2 min) and then at 14,000 rpm for 1 min for complete elution. RNA concentration was quantified with a Nanodrop One spectrophotometer (Thermo Fisher Scientific, Dreieich, Germany). 

The pH of the RNA column flow-through was adjusted by adding 480 µL molecular biology grade water and 40 µL binding buffer to the RNA column flow-through. The pH-adjusted sample was loaded onto the same RNA column (reused for protein purification) and centrifuged at 8000 rpm for 1 min. The column was washed twice with wash solution C. Finally, protein was eluted with 100 µL of protein elution buffer and 9.3 µL of protein neutralizer by centrifugation at 8000 rpm for 2 min. Protein concentration was determined with a Nanodrop One Spectrophotometer (Thermo Fisher Scientific, Dreieich, Germany). 

#### 4.3.7. Reverse Transcription and PCR Amplification (RT-PCR)

RT-PCR was performed with a commercial kit (One Taq RT-PCR kit NEB, Frankfurt am Main, Germany; E5310S) according to the manufacturer’s instructions. In brief, 50 ng of RNA was used to synthesize cDNA with random primer, M-Mul V reaction buffer, and Enzyme Mix (One Taq RT-PCR kit E5310S, NEB) using a PCR Thermocycler (Applied Bioscience/Thermo Fisher Scientific, Dreieich, Germany). First, the RNA was denatured at 70 °C in an RNase-free reaction tube containing 8 μL RNA/primer for 5 min. Then, the M-MuLV reaction mix (2X) and M-MuLV enzyme mix were added. For the RT negative control reaction, the M-MuLV enzyme mix was replaced with nuclease-free water. Tubes were incubated at 25 °C for 5 min and then at 42 °C for 60 min. Subsequently, the enzyme was inactivated at 80 °C for 5 min. An amount of 2 µL of diluted cDNA (1:1 with water) was used to perform PCR amplification using OneTaq Hot Start 2X Master Mix provided with the RT PCR Kit. An amount of 0.2 μM of rhodopsin, mGluR6, τGFP, and RIBEYE forward/reverse primers (Table 3) was used in a total reaction volume of 25 µL. The following amplification conditions for the reaction mix were used: initial denaturation at 95 °C for 30 s, then denaturation at 94 °C for 30 s, annealing at 58 °C for 30 s, and extension at 68 °C for 1 min. PCR amplification was repeated for 40 cycles and a final extension for 5 min at 68 °C was applied. PCR products were analyzed by 2% agarose gel electrophoresis. 

#### 4.3.8. Cloning and Sequencing of RT-PCR Products

For sequencing, the rhodopsin, mGluR6, τGFP, and RIBEYE RT-PCR bands were gel purified using the QIAEX II gel extraction kit (QIAGEN, Hilden, Germany). The purified PCR products were cloned blunt end in the pJET1.2 vector of the Clone JET PCR cloning kit (Thermo Scientific, K1231) using standard methods. XbaI/XhoI restriction digests were used to screen for recombinant positive clones. The T7 promoter primer TAATACGACTCACTATAGGG was used for sequencing of the recombinant plasmids. All cloned PCR products contained the correct inserts. 

## Figures and Tables

**Table 1:** Primary antibodies.

Antibody	Source	Reference	Dilution
RIBEYE(B)/CtBP2 (2D9), mouse monoclonal raised against the carboxyterminal 12 aa of mouse RIBEYE: KHGDNREHPNEQ	Lab-made	[38]	1:200 (IF)
HA, rat monoclonal IgG1 (3F10) raised against aa98–106 of influenza hemagglutinin (YPYDVPDYA)	Roche, 11867423001	[77]	1:300 (IF)
SV2, mouse monoclonal	Devel. Studies Hybrid. Bank; Iowa City, IA, USA	[78]	1:100 (IF)
Cre, mouse monoclonal (2D8)	Millipore, MAB3120	[79]	1:300 (IF)
Cre, rabbit polyclonal	LSBio LifeSpan Biosci LS-C179954/64865	[80]	1:300 (IF)

IF, immunofluorescence microscopy.

**Table 2:** Secondary antibodies.

Antibody	Source	Dilution
Donkey anti-mouse IgG (H + L) Alexa 568	Invitrogen; Karlsruhe, Germany; A-10037	1:1000 (IF)
Chicken anti-rabbit IgG (H + L) Alexa 568	Invitrogen; Karlsruhe, Germany; A-10042	1:1000 (IF)
Donkey anti-rat IgG (H + L) Alexa 488	Invitrogen, Karlsruhe, Germany; A21208.	1:1000 (IF)

IF, immunofluorescence microscopy.

**Table 3:** Primers for RT-PCR.

Primer 1 (RIBEYE/Cre forward primer)	GTGAGGCAAAGTTACCGTGAGT
Primer 2 (RIBEYE/Cre reverse primer)	GACCGGCAAACGGACAGAAG
Primer 3 (Rhodopsin forward primer)	GTGCCCTTCTCCAACGTCAC
Primer 4 (Rhodopsin reverse primer)	GAAGCCCTCGAGATTACAGCCT
Primer 5 (mGluR6 forward primer)	AGTGTGACATGTCTGATCTGTC
Primer 6 (mGluR6 reverse primer)	TACTTGGCGTCCTCTGAGTTC
Primer 7 (GFP forward primer)	GGACGACGGCAACTACAAGACC
Primer 8 (GFP reverse primer)	CACGAACTCCAGCAGGACCATG

## Data Availability

All data are presented in the manuscript.
